# Emerging Molecular Prospective of SARS-CoV-2: Feasible Nanotechnology Based Detection and Inhibition

**DOI:** 10.3389/fmicb.2020.02098

**Published:** 2020-10-20

**Authors:** Sushmita Patra, Rout George Kerry, Ganesh Kumar Maurya, Bijayananda Panigrahi, Swati Kumari, Jyoti Ranjan Rout

**Affiliations:** ^1^Department of Biotechnology, North Orissa University, Baripada, India; ^2^Department of Biotechnology, Utkal University, Bhubaneswar, India; ^3^Zoology Section, Mahila Mahavidyalaya, Banaras Hindu University, Varanasi, India; ^4^School of Biotechnology, Kalinga Institute of Industrial Technology, Bhubaneswar, India; ^5^School of Biological Sciences, AIPH University, Bhubaneswar, India

**Keywords:** autophagy, COVID-19, genetics and epigenetics, inflammasome, nanobased diagnosis, nanobased therapeutics, SARS-CoV-2 pathogenesis

## Abstract

The rapid dissemination of SARS-CoV-2 demonstrates how vulnerable it can make communities and is why it has attained the status of global pandemic. According to the estimation from Worldometer, the SARS-CoV-2 affected cases and deaths are exponentially increasing worldwide, marking the mortality rate as ∼3.8% with no probability of its cessation till now. Despite massive attempts and races among scientific communities in search of proper therapeutic options, the termination of this breakneck outbreak of COVID-19 has still not been made possible. Therefore, this review highlights the diverse molecular events induced by a viral infection, such as autophagy, unfolded protein response (UPR), and inflammasome, illustrating the intracellular cascades regulating viral replication inside the host cell. The SARS-CoV-2-mediated endoplasmic reticulum stress and apoptosis are also emphasized in the review. Additionally, host’s immune response associated with SARS-CoV-2 infection, as well as the genetic and epigenetic changes, have been demonstrated, which altogether impart a better understanding of its epidemiology. Considering the drawbacks of available diagnostics and medications, herein we have presented the most sensitive nano-based biosensors for the rapid detection of viral components. Moreover, conceptualizing the viral-induced molecular changes inside its target cells, nano-based antiviral systems have also been proposed in this review.

## Introduction

Pneumonia, an infection of the lungs, is sometimes associated with severe epidemics due to specific viral infections as evidenced in 2002, 2012, and most recently in 2019 due to severe acute respiratory syndrome (SARS), Middle East respiratory syndrome (MERS), and COVID-19, respectively (Christian et al., 2004; Assiri et al., 2013; Sahin et al., 2020). COVID-19 disease caused by severe acute respiratory syndrome coronavirus-2 (SARS-CoV-2) was declared as a global pandemic by the World Health Organization (WHO) on March 11, 2020 (WHO, 2020d). Commencement of this viral infection with pneumonia-like symptoms was reported to be associated with a seafood market in Wuhan, China in December, 2019 (Adhikari et al., 2020, Holshue et al., 2020). Afterward, the movement of people during Chinese New Year became a ground for its escalation throughout China and then throughout the globe. Rapid investigations of the clinical manifestation associated with prevailed infection revealed a similar pattern to that of SARS and MERS infections. Severity of COVID-19 infection is associated with some risk factors such as age, obesity, and other specific comorbidities including high blood pressure, pregnancy, diabetes, asthma, and impaired immunity (Guo G. et al., 2020; Petrakis et al., 2020). Within 6–7 months of its emergence, the virus spread swiftly worldwide, affecting 213 countries and territories with around 18.0 million confirmed cases being reported (Worldometer, 2020). Thus, it has provoked a standstill of life in both developed and developing countries. There is a report of about 18,055,630 COVID-19 cases worldwide with a mortality rate of ∼3.8% (August 2, 2020), and the cases are expected to increase further (Worldometer, 2020).

There is an urgent need for rapid and efficient methods for SARS-CoV-2 diagnosis and to overcome the present viral outbreak. Currently available diagnostic kits detect the infection from within around 8 h to ≤15 min (Carter et al., 2020). The viral molecular components, specifically the detection of ORFs encoding pp1ab, nsp 14, N protein, S protein and RNA-dependent RNA polymerase (RdRP) protein, through real time PCR (RT-PCR) methods are being considered. Additionally, the serum-based techniques that employ viral antibodies take less time for diagnosis in comparison to RT-PCR methods. However, due to the lack of committed anti-viral therapeutics against COVID-19, previously known antiviral strategies involving experimental conventional antiviral drugs used conjointly are being used to treat the symptoms associated with the infection. These drugs are conventionally used in the treatment of other deadly viral/protozoal infections such as HIV/AIDS, influenza virus, Ebola virus, etc. Due to their side-effects, application of these drugs against this contagious viral infection has become a matter of debate (Corman et al., 2020; Udugama et al., 2020).

These limitations in SARS-CoV-2 detection and effective inhibition could be neutralized by the implementation of nanotechnology. Previously, Gold (Au), Silver (Ag), Graphen, Carbon dots, and Silica (Si) nanoparticles (NPs) have been used for inhibition of a number of viruses (Kerry et al., 2019; Kondel et al., 2019; Lauster et al., 2020). Further, the currently available antiviral drugs barely utilize the emerging molecular intracellular pathways, like autophagy, inflammasome, genetics, and epigenetics, for viral inhibition (Prentice et al., 2004a; Tang et al., 2009; Maier and Britton, 2012; Schafer and Baric, 2017; Fung et al., 2020). In the present review, a detailed outline of the emerging molecular intracellular pathways of SARS-CoV-2 and their significant interactions with host cells are briefly described. Additionally, different types of nanotechnological platforms with possible applications in viral detection and inhibition are also narrated. Specifically, we discuss how the change in the optical property of AuNPs could be exploited in SARS-CoV-2 detection. And how viral antibody conjugated carbon-NPs-coated electrochemical strip could be used for SARS-CoV-2 detection is described from the knowledge and inspiration of previously published and relevant research in virus detection and inhibition (Galluzzi et al., 2017; Chen et al., 2018; Jazayeri et al., 2018; Chowdhury et al., 2019; Siu et al., 2019; Conti et al., 2020; Jena et al., 2020). Moreover, engineering of a cellular-targeted nuclear delivery of antiviral drugs in conjugation with an antisense siRNS or antisense oligonucleotide is also presented for simultaneously sustaining host cell survival and viral inhibition. The antisense siRNS or antisense oligonucleotide can target molecular regulation of autophagy, inflammasome, or endoplasmic reticulum stress of the infected cell, resulting in either inhibiting the viral process within the host cell or armoring the host cell to combat viral infection. Overall, the review could boost scientific innovation to develop an effective and smart diagnostic tool and nanotechnology-based SARS-CoV-2 inhibiting agent.

## SARS-CoV-2 Basic Information

Coronaviruses belonging to the family Coronaviridae are represented by four genera – alpha (α), beta (β), gamma (γ), and delta (δ) – as per the nature of their genetic reservoir, the kind of host, and mode of recombination (Wertheim et al., 2013). The viruses are mostly zoonotic and infect many species of animals, such as camels, cats, bats, and cattle, but have now transferred swiftly to humans. So far, seven such types of human-to-human transmitted coronaviruses have been reported, and these are denominated as 229E and NL63 of genera α-coronavirus and OC43, HKU1, SARS-CoV, MERS-CoV, and SARS-CoV-2 of genera β-coronavirus (Chu D.K.W. et al., 2020). Among all these, SARS, MERS, and COVID-19 are known to be highly pathogenic with a high fatality rate unlike others that are associated with mild upper respiratory tract infections. The world previously had evidenced two coronavirus epidemics caused by SARS-CoV and MERS-CoV in the years of 2002 and 2012, respectively.

The coronavirus is again in the spotlight and has raised alarm across the world after its first official report from Wuhan, China. This time the 2019-CoV was christened as SARS-CoV-2 as it is genetically similar to SARS-CoV, and the disease was named COVID-19 (Coronavirus disease 2019) by WHO. Later on, Andersen et al. (2020) suggested that though 2019-CoV holds a ∼96% genetic similarity with RaTG13 bat virus (*Rhinolophus sinicus*), it has overcome the species-to-species barrier through natural selection (Song et al., 2020). The transmission of the virus to humans occurs through an encounter with infected respiratory aerosols, feces, or through direct contact with the contaminated persons (Xiao et al., 2020). Analysis of several suspected cases indicated that the median incubation period of the virus is around 4.5–5.1 days and symptom onset time is about 11.5–14.5 days (Lauer et al., 2020; Wang X. et al., 2020). The infected persons can be categorized as either symptomatic or asymptomatic, but both have the ability of virus transmission (Gao et al., 2020). However, patient’s clinical manifestations include pneumonia, non-productive cough, fatigue, fever, acute respiratory distress syndrome (ARDS), standard or decreased leukocyte count, and organ dysfunction, ultimately leading to death (Chu D.K.W. et al., 2020). Due to the novelty of the virus, till now no such specific drugs/vaccines have been formulated to overcome COVID-19, however investigation is under process. Thus, acknowledging the gravity of the situation, an effort has been made to collate novel ideas from different literature, with a hope that this collective review may partially help scientists to find some innovative solutions to shield or combat this deadly disease.

## Structure

Belonging to the family of Coronaviridae in the order of Nidovirales, SARS-CoV-2 embraces a 30 kilobases long, single positive-stranded RNA genome, endowed with 14 open reading frames (ORFs) and encoding 27 proteins (Wu A. et al., 2020; Zu et al., 2020). The genome is protected by a long, coiled helical capsid of 10-20 nm in diameter, which is further surrounded by an envelope. Whole genome sequence analysis has revealed four main structural proteins, spike (S), envelope (E), membrane (M), and nucleocapsid (N) protein, which are encoded by ORF2, ORF4, ORF5, and ORF9, respectively, present at 3′-terminus constituting about one-third of the viral genome. The transmembrane spike glycoprotein (150 kDa) engrosses into the viral envelope, and the protruding peplomers illustrate a crown-like appearance of the virus. Entry into the host cell is mediated by the attachment of homotrimeric S protein that is cleaved by host cell protease and generates two non-covalently attached functional subunits (Coutard et al., 2020). S1 subunit is a receptor binding domain and determines cellular tropism in several host virus range, while the S2 subunit mediates fusion with the cell membrane (Astuti and Ysrafil, 2020). Both SARS-CoV and SARS-CoV-2 share 91.5% homology in their S protein sequences, but the cleavage site (QTQTNSPRRARSVASQSIIA) is unique to SARS-CoV-2 (Yoshimoto, 2020). The ORF4-encoded smallest E protein is responsible for viral assembly, release and, thus, maturation. Beneath the spike lies an M protein that provides shape and stability to the virus structure interacting with the N protein associated with RNA. According to Fehr and Perlman (2015), the binding of the N protein to the viral genome represents the beads-on-a-string type conformation. N protein along with M protein mediates viral packaging and acts as a suppressor of antiviral RNA interference in SARS-CoV-2 (Mu et al., 2020). In addition to these structural proteins, eight more protein coding genes are present near 3′ terminus of the genome, known as accessory proteins: 3a, 3b, p6, 7a, 7b, 8a, 9b, and orf 14 (Wu A. et al., 2020). The 15 non-structural proteins (nsp1-10 and nsp12-16) are produced through the cleavage of polyproteins pp1ab and pp1a expressed by the orf1ab and orf1a gene constituting two thirds of the viral genome at its 5′end (Mirza and Froeyen, 2020).

The first non-structural protein (nsp1), also christened as leader protein, has been reported to degrade host mRNA interacting with the 40S ribosomal subunit in SARS-CoV. The virus expresses two proteases, 3-chymotrypsin-like protease (3CLpro) and papain-like protease (PLpro), which are encoded by nsp5 and nsp 3, respectively (Kim et al., 2020). The 3CLpro, with a catalytic dyad defined by His 41 and Cys145, has 11 cleavage sites at the C-terminus (releasing Nsp4–Nsp16), whereas PLpro cuts at three other sites toward the N-terminus, mainly at 181–182, 818–819, and 2763–2764, generating a total of 15 non-structural proteins (Ton et al., 2020; Wu A. et al., 2020). The nsp12 codes for RdRp and replicates RNA from RNA templates and nsp13 for helicase (Lung et al., 2020). The viral nascent RNA is capped at 5′end and is catalyzed by the enzyme guanine-N7-methyltransferase that is coded by nsp14. Additionally, the modification of this cap structure is executed by nsp16-encoded enzyme 2′-*O*-methyl transferase (Iftikhar et al., 2020). Though the receptor-binding domain in the S protein of SARS-CoV-2 is homologous with that of SARS-CoV, the amino acid varies at some key residues. A significant difference is the position of RBD in their respective down conformation. In the case of SARS-CoV, the RBD remains packed against the N-terminal domain while it remains inclined closer to the central cavity of the trimer in SARS-CoV-2 (Wrapp et al., 2020). Irrespective of these variations, the amino acids at the interface residues of the SARS-CoV-2 RBD include Leu455, Phe456, Tyr473, and Gln493 in place of Tyr442, Leu443, Phe460, and Asn473, respectively, in SARS-CoV RBD (Yan et al., 2020).

## Mechanism of Infection in Human

Though the receptor binding domain of SARS-CoV is similar to that of SARS-CoV-2, spike proteins of the latter have a strong affinity for the angiotensin-converting enzyme 2 receptor (ACE2) expressed abundantly on the human type II alveolar epithelial cells (AT2) of the lung, urothelial cells of the bladder, heart, and kidney, and enterocytes of intestinal cell surfaces (Sureda et al., 2020; Xu et al., 2020). SARS-CoV-2 can infect type I and type II pneumocytes as well as alveolar macrophages (Chu D.K.W. et al., 2020). The integral membrane protein ACE2, which accommodates metallopeptidase domain (PD) and collectrin-like domain (CLD), controls vasoconstriction and blood pressure, and plays a vital role in antiproliferation through the maturation of angiotensinII (Ang II) to angiotensin 1-7 [Ang(1-7)], serves as an antagonist of type 1 angiotensin receptor (AT2-R) (Tikellis and Thomas, 2012; Fandiño et al., 2018). Researchers have provided dismaying evidence of T-cell infection by SARS-CoV-2. It indicates the capability of the virus in binding receptors other than ACE2 as the expression of ACE2 receptor in T-cells is low (Wang Q. et al., 2020).

The 31st amino acid lysine present in ACE2 receptors of the host is reported to be identified by the glutamine amino acid of residue 394 in the receptor-binding domain (RBD) of SARS-CoV-2. The 394 residue corresponds to residue 479 with a similar amino acid in SARS-CoV (Zhang H. et al., 2020). Structurally RBD of SARS-CoV-2 consists of a core region and a receptor binding motif (RBM). The end region of RBD that sets up contact with the ACE2 receptor is characterized by three variable loop regions, or contact regions: CR1, CR2, and CR3. Analysis of the RBD-ACE2 complex demonstrates a hydrogen bonding pattern between the CR3 loop region and ACE2 binding surface, whereas CR1 (N-terminal loop region) and CR2 (central region) strengthen binding through a hydrophobic interaction at the dimer interface (Wang Y. et al., 2020). Ingress into the host cell is mediated by the spike protein of the virus through the attachment of the S1 subunit on to the target cell surface. Research indicates that the C-terminal domain (CTD) of the S1 subunit in the spike protein of SARS-CoV-2 establishes more interactions with the hACE2 receptor, showing a binding affinity 10–15 times greater than SARS-CoV RBD (Wang Q. et al., 2020). Further, the priming of the S protein is essential for the fusion of the virus to human lung cells, facilitated by cellular proteases of the host (Letko et al., 2020). As a result, the cleavage of S protein at S1–S2 and S2’ sites occur and the whole process is driven by the S2 subunit of the spike protein (Hoffmann et al., 2020). In the case of SARS-CoV-2, the cellular serine protease, TMPRSS2, expressed in ACE2^+^ human pulmonary cells recognizes the “RRER” sequence at S1-S2 site and acts as a priming agent of the S protein (Guo Y.-R. et al., 2020; Wang Q. et al., 2020). As mentioned earlier, due to the higher homology of SARS-CoV-2 with SARS-CoV, it would be speculated that they follow a similar entry pathway.

The fusion is followed by the uncoating and subsequent release of 30,000 base pairs of long +sRNA genomes, from which replicase genes (two thirds of the viral genome), encompassing ORF1a and ORF1b, are first translated into replicase polyprotein pp1a and pp1ab (Cascella et al., 2020). Translation initiates at ORF1a, and ribosomal shifting ensures its uninterrupted expression up to ORF1b. The nsp3 and nsp5 present within ORF1a-ORF1b are responsible for autoproteolytic cleavage of large polyprotein as it encodes two viral proteases PLpro and Mpro (Gordon et al., 2020). Consequently, 16 non-structural proteins and RdRp are the products of this autoproteolytic activity. With the help of this Nsp12-encoded RdRp enzyme, a -sRNA is synthesized using the +sRNA as a template. Newly synthesized –sRNA serves as the template for the synthesis of the viral +sRNA genome. The nsp3, nsp4, and nsp6 encode several integral multi-spanning membrane proteins, playing an essential role in anchoring the replication-transcription complex (RTC) to the intracellular membranes. The other one third of the genome is discontinuously transcribed into a set of subgenomic mRNAs which are translated into viral structural proteins S, E, M, and N proteins, as well as accessory proteins. Among these structural proteins, N protein is synthesized within the cytoplasm and encircles the genomic RNA to construct the virion, whereas the other three proteins, S, E, and M are translated and post-translationally modified inside the endoplasmic reticulum (Fung and Liu, 2014). Further assembly and maturation of the virion occurs inside the ER-Golgi intermediate compartments (ERGIC) because, after folding, the assigned proteins (S, M, and E proteins) are transported to ERGIC. Mature virus particles encapsidated inside the smooth vesicle are released upon fusion with the host cell plasma membrane. Previous findings suggest that during viral assembly, cell lines expressing TMPRSS2 protease tend to express spike proteins on the surface of their membrane, inducing attachment with the uninfected neighboring cells. Their fusion brings about the development of a large multinucleated cell, called syncytium (Matsuyama et al., 2020).

## Autophagy

Autophagy is a self-cleaning biological process that obliterates the deleterious and malformed proteins and damaged organelles to regulate cellular homeostasis even under stress conditions. The unwanted cell components targeted for elimination are enclosed in a double membrane vesicle that ultimately fuses with lysosome for degradation of those enclosed components. Though the process is associated with cellular defense and survivability, it is also exploited by a few viruses to increase their infectious potency (Rozières et al., 2017). Such viruses slightly block this lysosomal degradation pathway and defend themselves by exploiting it to support their replication and virulence. Among them, CoV is reported to induce double-membrane vesicles’ (DMV) formation in the parallelism of autophagosome that originates through the interaction of several proteins coded by autophagy related genes (ATG) (Prentice et al., 2004a). In a follow-up study, researchers demonstrated the colocalization of RTC with autophagic proteins LC3 (microtubule-associated protein light chain 3) and Atg12 at DMV (Prentice et al., 2004b). In general, this self-digesting process involves three steps- initiation, elongation, and fusion – which are regulated by 16 autophagy-related proteins (Atg proteins) and two ubiquitin-like conjugation systems. In brief, two complexes, one including class III phosphatidylinositol 3-kinase Vps34, Atg14, and the Atg6/Beclin1 complex while the second complex consists of serine/threonine kinase Atg1 associated with Atg8, LC3, GATE-16, and GABARAP in mammals, contribute together to generating phagophore (Maier and Britton, 2012). The expansion of phagophore and formation of autophagosome needs the recruitment of two-ubiquitin-like complexes, such as Atg12-Atg5-Atg-16 and Atg4B, Atg3, and Atg7, which facilitate the LC3 lipidation that is considered as a hallmark of autophagy. Complete formation of the autophagosome leads to the subsequent dissociation of the Atg16-Atg5-Atg12 complex and further fusion with lysosome is assisted by VPS15, UVRAG, VPS34, and Beclin1 complex. Cottam et al. (2011) demonstrated the autophagy process to be induced by nsp6-encoded viral replicase protein in an infectious bronchitis virus (IBV) strain of CoV. Although CoV induces autophagy, several studies also indicate that it exhibits incomplete autophagy formation due to impaired fusion of autophagosomes with the lysosome, which is triggered by nsp3 coded PLpro in SARS-CoV, MERS-CoV, and membrane-associated papain like protease PLP2 (PLP2-TM) in HCoV-NL63 (Chen et al., 2014; Yang and Shen, 2020). Association of PLP2-TM with LC3 and Beclin1 further enhances the interaction between Beclin1 with STING, consequently suppressing the expression of antiviral interferons. Adaptation of the virus to this incomplete autophagy process prevents degradation of their components, thus blocking them from the innate antiviral immunity. Schneider et al. (2012) signifies that SARS-CoV replication is not hampered by the downregulation of ATG7 or ATG5, key autophagy proteins also involved in autophagosome biogenesis. Analysis of data from various studies conclude that the inhibition of autophagy does not impair the CoV replication and infection process, but rather that activation of autophagy and its subsequent maturation might lead to the destruction of viral components (Carmona-Gutierrez et al., 2020). Therefore, it can be assumed that the activation of the autophagy process might be an impactful step in inhibiting viral replication, as both SARS-CoV and SARS-CoV-2 share somewhat similar infection processes, like the involvement of host cell receptor ACE2.

## Endoplasmic Reticulum Stress and Apoptosis

**FIGURE 1:**
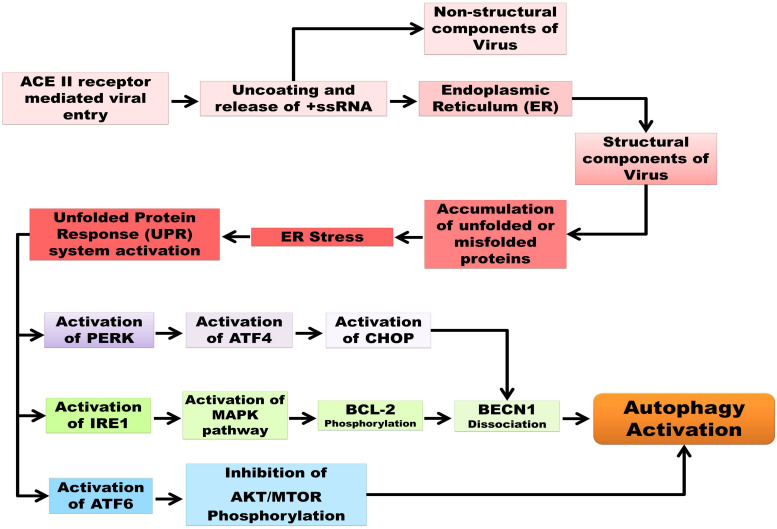
Schematic representation of autophagy and UPR pathways as well as their interconnection in SARC-CoV-2 pathogenesis. Unfolded protein response (UPR), protein kinase R-like endoplasmic reticulum kinase (PERK), serine/threonine-protein kinase/endoribonuclease inositol-requiring enzyme 1 (IRE1), activating transcription factor 6 (ATF6), activating transcription factor 4 (ATF4), C/EBP homologous protein (CHOP), mitogen-activated protein kinase (MAPK)/c-Jun N-terminal kinase (JNK) pathway, B-cell lymphoma 2(BCL2), Beclin 1 (BECN1), mechanistic target of rapamycin (MTOR), serine/threonine-specific protein kinase (AKT).

Various analyses have concluded that CoV infection (not specifically 2019-nCoV) is directly associated with the endoplasmic reticulum stress induction in host cells due to three specific viral mechanisms: DMV formation, post-translational modification of viral structural proteins, and lipid depletion during the exocytosis of virions (Fung and Liu, 2014). Snijder et al. (2006) demonstrated the co-localization of SARS-CoV non-structural proteins along with an ER marker, protein disulphide isomerase (PDI). Two endosome cargos, mannosidase α-like 1 (EDEM1) and osteosarcoma amplified 9 (OS-9), have been shown to colocalize with the RTCs during mouse hepatitis virus (MHV) infection, as observed by Reggiori et al. (2010). After a couple of studies, a model has been proposed regarding the sequestration of COPII-independent endosomes exported from ER by CoV for its DMV formation, suggesting ER as the origin of DMVs. Coronavirus replication and assembly lead to great ER-stress, as the viral trimeric spike glycoprotein undergoes glycosylation at the S2 domain, maturation, and further assembly inside ER, mediated by calnexin-like chaperons (Fukushi et al., 2012). According to Nal et al. (2005), not only the S protein but also the M protein of β-coronavirus undergoes O-linked glycosylation inside ER. As mentioned before, ERGIC is the site of virion assembly; therefore, budding or exocytosis of mature virions causes the cutback of lipid from ER. The translation of viral proteins inside ER leads to the accumulation of unfolded and misfolded proteins inside ER lumen that overwhelms ER capacity. For upholding the stability of ER during CoV infection, the signaling pathways, called unfolded protein response (UPR), are activated by interacting with its three branches: protein kinase RNA-activated (PKR)-like ER protein kinase (PERK), activating transcription factor 6 (ATF6), and inositol-requiring enzyme 1 (IRE1). Earlier studies have shown phosphorylated PERK, PKR, and eIF2α in SARS-CoV infected cells and suggested the activation of PKR by its infection might lead to apoptosis (Krähling et al., 2009). Moreover, Deng et al. (2017) has pointed out the possibility of PKR suppression is due to the action of nsp15-coded endonuclease. Shi et al. (2019) also indicated that ORF-8b expression induces the upregulation of CHOP and UPR proteins, which together promotes cell death by forming protein aggregates relying on its amino acid valine at 77 positions. Although the ORF-8b of SARS-CoV has less homology with that of SARS-CoV-2, the maturation of viral proteins occurring inside the ER might increase the workload, causing ER stress and subsequent apoptosis (Fung et al., 2020). Furthermore, the PERK and IRE1 signaling pathways are common to both the cellular events of ER stress and autophagy, which justify that these processes are inter-connected via the UPR system (Shojaei et al., 2020). The accumulated unfolded and misfolded proteins subsequently increase ER stress and, to combat it, the cytosolic ubiquitin-proteasomes level increases (Boga and Coto-Montes, 2020). Before apoptosis, cells activate autophagy to degrade the protein aggregates for their survivability (Yorimitsu et al., 2006). But the induction of incomplete autophagy upon SARS-CoV-2 infection generates DMV that in turn supports viral replication. Activation of autophagy by PERK and IRE1 branches are interconnected where activated DNA damage-inducible transcript 3 (DDIT3), also known as CCAAT/enhancer-binding protein homologous protein (CHOP), facilitates the dissociation of beclin1 (BECN1) from B-cell lymphoma 2 (BCL2). The activation of CHOP expression is induced via activating transcription factor (ATF4) stimulation by PERK. Similarly, evidence also suggests that the IRE1 pathway helps to trigger the phosphorylation of BCL2 through the mitogen-activated protein kinase (MAPK/JNK) pathway, which in turn enables beclin 1 (BECN1) to induce autophagy (Kapuy et al., 2014). Besides this, ER stress mediated the downregulation of the cell growth regulator mechanistic target of rapamycin (MTOR), which activates autophagy by inhibiting the serine/threonine-protein kinase (AKT) in the AKT-MTOR signaling pathway regulated by the ATF6 branch; the whole process of the autophagy/UPR pathway and their interconnections are represented in Figure 1 (Rashid et al., 2015; Bello-Perez et al., 2020; Sureda et al., 2020).

## Inflammasome

The inflammasome in multiprotein complexes is accountable for the activation of inflammatory reactions, having a role in promoting antiviral innate immune response and the spreading of viral infection. Among all inflammasomes, the NOD-like receptor (NLR) family pyrin domain-containing 3 (NLRP3) inflammasome is mostly involved in inflammation, and pyroptosis are ensured due to viral infections. As reports suggest, viruses exploit this phenomenon to escalate their infection rapidly and to evade the host’s immune system by facilitating the expression of IL-1β through the activation of NLRP3 inflammasome. The ion-redistribution, mitochondrial damage, and lysosomal disruption induced by the viroporins (ORF3a, ORF8b, and E protein) of SARS-CoV account for caspase-1 activation accelerating NLRP3 inflammasome stimulation (Shah, 2020). SARS-CoV E protein and ORF-3a promote NF-κB mediated pro-IL-1β gene transcription, IL-1β secretion, and NLRP3 expression (Freundt et al., 2010; Nieto-Torres et al., 2015). Recent evidence has pointed out the ORF3a-mediated activation of NLPR3 inflammasome in SARS-CoV-1 infected cells promotes TRAF3-dependent facilitation of polyubiquitination of apoptosis-associated speck-like protein (ASC). The interaction of trio-ORF3a with TRAF3 along with ASC leads to ASC speck formation (Siu et al., 2019). ASC, carrying the inactive procaspase-1 along with the caspase activation and recruitment domain, constitutes the adapter component of NLRP3. As per the pathogenesis of SARS-CoV-2, the lung inflammation indicates the interaction of the virus with toll-like receptor (TLR), which promotes the expression of pro-IL-1β and the release of IL-1β. The secretion of inflammatory cytokines further triggers the expression of other downstream effectors involved in inflammation, including tumor necrosis factor (TNF), interleukin 6 (IL-6), leukotrienes, and prostaglandins (Mangan et al., 2018). Activation of NLRP3 inflammasome and caspase-1 induces expression of non-canonical caspases, including caspase-4, caspase-5, and caspase-11, which promote pore formation on cell membranes by causing the release of gasdermin-D. Consequently, a massive influx of water and Na^+^ ions result in excessive swelling of cells, leading to cell membrane rupture or pyroptosis (Yap et al., 2020). However, SARS-CoV E protein shares a 95% homology with its counterparts in SARS-CoV-2, whereas ORF3a has only 72%, so it can be extrapolated that the E protein of SARS-CoV-2 might have a role in the activation of inflammasome (Fung et al., 2020). Considering the role of E protein in the activation of NLRP3 inflammasome, it can be affirmed that targeting the viral E protein would extricate the host cell from the detrimental effects of the inflammasome.

## Immune Response of SARS-CoV-2

Invasion of a virus surges both innate and adaptive immunity. Further destruction of virus-infected cells elicits local macrophages and monocytes to migrate and release cytokines that trigger T and B cells. Viral infection causes the release of diverse pathogen-associated molecular patterns, such as viral RNAs, proteins, and ASC oligomers, which are recognized by toll-like receptors (TLRs) and RIG-like receptors (RLRs), a kind of pattern-recognition receptors (PRRs) that are located in cytosolic or endosomal compartments (Park and Iwasaki, 2020). Activation of alveolar macrophages and lymphocytes trigger inflammation as well as the release of pro-inflammatory cytokines and chemokines in an increased level (Zhong et al., 2020). Reports suggest that this cytokine storm, including IFNγ-induced protein-10 (IP10), macrophage chemoattractant protein1 (MCP1), IL-6, tumor necrosis factor α (TNFα), granulocyte colony-stimulating factor (GCSF), and macrophage inflammatory protein 1α (MIP1α), are associated with severe clinical outcomes in COVID-19 patients (Huang et al., 2020; Tay et al., 2020). Higher concentrations of cytokines and pro-inflammatory cytokines cause acute respiratory distress syndrome (ARDS), which is a major factor of death in cases of the recent coronavirus infection. To evade the innate immune system, the virus has developed diverse strategies such as avoiding PAMPs recognition by PRRs and the inhibition of interferon production and interferon-signaling pathways. Interferon, being a part of the innate immune system, exhibits antiviral activity (Noh, 2020). Viral infection induces both type 1 and type III interferons that further execute a plethora of effector mechanisms against viruses. In the case of viral infection, detection of virus-associated PAMPs by intracellular PRRs subsequently activates transcription factors, mainly interferon regulator factors (IRFs) and nuclear factor κB (NF-κB). The activation of IRFs evoke an antiviral response by enhancing the expression of type I and type III interferons, as well as IFN-stimulated genes (ISGs). Type 1 IFN interaction with its receptor initiates a signaling cascade through phosphorylation and subsequent activation of transcription factors. The transcription factors are signal transducer and activators of transcription 1 (STAT1) and STAT2 which, along with IRF9, actuate ISGs’ expression via IFN-stimulating gene factor 3 (ISGF3) intermediate complex formation (Blanco-Melo et al., 2020). As a result, virus replication is interrupted and viral resistance is developed, ensuring the induction of the adaptive immune system. The 5′ capping mechanism performed by nsp14 and nsp16 in SARS-CoV-2 enables viral RNA to mimic host RNA, that in turn can prevent its detection by RLR receptors. SARS-CoV-1 infection promotes type 1, type II, and type III IFNs’ production while SARS-CoV-2 is not reported to induce their expression, which leads to insignificant activation of the innate immune system. An asymptomatic phase of COVID-19 in patients could arise due to this low activation of the immune system (Chu H. et al., 2020). In SARS-CoV-1, ORF3b, ORF6, nsp1, nsp3, and two structural proteins, N and M, act as antagonists to IFN signaling (Totura and Baric, 2012). As per the sequence analysis study, SARS-CoV-2 has a high sequence homology with nsp1 and other structural proteins of SARS-CoV-1 but differs greatly with respect to the other above-mentioned genomic regions. Recent studies suggest that the 22 amino acid long ORF-3b protein of SARS-CoV-2 can significantly inhibit IFN activation (Konno et al., 2020) however, SARS-CoV-2 susceptibility to type 1 interferon is still under scrutiny.

## Genetics and Epigenetics

Pathogenesis of a virus can be ascertained through the impact of diverse cytopathic effects and through genetic and epigenetic changes. Human coronaviruses, including HCoV-229E, NL63, HCoV-OC43, and SARS-CoV, upon infection are reported to bring out transcriptome changes in the host cell. In the case of SARS-CoV, microarray analysis reveals that around 164 genes are altered; out of these, 17 are downregulated, while 49 genes are known to be upregulated. Among the upregulated genes, about 11 genes, like proapoptotic, 4 genes- phospholipid scramblase 1 (PLSCR1), thrombospondin 1 (THBS1), early growth response 1 gene (EGR1), and plasminogen activator inhibitor 1 (PAI1/SER-PINE1), are associated with the procoagulant pathway. In addition, 32 genes, including transforming growth factor β2 (TGF-β2), IL-8, chemokines CXCL-1, -2, -3, -5, -6, -10, tumor necrosis factor α (TNF-α), and many more, are also stimulated by inflammation and immune response (Tang et al., 2009). Thus, CoV infection conciliates massive genetic changes in the host cell. Epigenetics, the interface between the environment and the genome, deals with alterations in gene expression patterns through various modifications in a gene, such as DNA methylation, chromatin remodeling, and histone modifications, without altering the genome sequences. A broad range of studies have explained the virus-mediated host’s epigenetic modulation that supports their replication and pathogenesis by evading the host’s antiviral immune system. Many recent studies demonstrate that CoV enhances its virulence potency by manipulating IFN-stimulated gene (ISG) histone modification, resulting in the delayed expression of ISG effectors validated in both SARS-CoV and MERS-CoV (Schafer and Baric, 2017). The molecular mechanism behind the upregulation of several NF-κB target genes in SARS-CoV-infected cells suggests the underlying post-translational modification of several proteins to phosphorylation, ubiquitylation, and subsequent proteasomal degradation of the inhibitor of NF-κB (IκB)-like proteins (Smits et al., 2010; Poppe et al., 2017). However, these genetic and epigenetic modifications of the host infected with SARS-CoV-2 have not yet been properly investigated. Currently available research is mostly associated with epigenetic modifications of ACE2 receptors. Available research evidence suggests that oxidative stress induced by SARS-CoV-2 infection leads to the overexpression of ACE2 due to its hypomethylation on the X chromosome (Pruimboom, 2020). Commonly, the lowest expression of ACE2 receptor in leukocytes and neurons is associated with its hypermethylated state, while in lung epithelial cells, the gene is hypomethylated (Corley and Ndhlovu, 2020). In short, these findings describe the role of epigenetics in age-related fatality of COVID-19, whereas ACE2 hypomethylation demonstrates a direct proportionality with aging as well as greater viremia. Therefore, present investigations are more focused on targeting the epigenetic modifications of host’s ACE2 receptors (Mueller et al., 2020; Sawalha et al., 2020).

## Available Detection and Inhibition Strategy of SARS-CoV-2

Early and rapid diagnostic techniques are imperative in the management of the SARS-CoV-2 outbreak. But presently available diagnostic kits, for example, PCR based techniques, serum-based tests, and the most recently developed non-invasive tests, take a detection time of around 2–8 h, 15–60 min, and ≤15 min, respectively (Carter et al., 2020). The viral molecular components, specifically ORF 1ab and nucleocapsid gene N, ORF1b-nsp 14, S gene, and RdRP gene, are currently being targeted for the detection of SARS-CoV-2 infection through the RT-PCR methods. Presently, serum-based diagnostic tests are preferred overt RT-PCR based diagnostic tests due to their shorter time frame of detection (Table 1A).

**TABLE 1A:** List of different diagnostic test available for detection of SARS-CoV-2.

Sl. No.	Name	Developing organization	Mode of detection	Mechanism of detection	References
***(A) Nucleic acid-based test***					
1	New coronavirus nucleic acid determination kit	Chinese National Institute for Viral Disease Control and Prevention, China	Real-time fluorescence RT-PCR method	Detecting the novel coronavirus with RT-PCR using primers against ORF 1ab and nucleocapsid gene N, and fluorescent probes	CDCP, 2020b; Sheridan, 2020; WHO, 2020a
2	CDC 2019-nCoV real-time reverse transcriptase PCR diagnostic panel	US Centers for Disease Control and Prevention (CDC), United States	Real-time RT-PCR method	Real-time RT-PCR using primers against three targets in nucleocapsid gene N, along with Human RNase P gene as a positive control	CDCP, 2020a, b; Sheridan, 2020; Udugama et al., 2020; WHO, 2020a
3	Real-time reverse transcriptase PCR assays	University of Hong Kong, Hong Kong	Real-time RT-PCR method	Two single-step quantitative real time RT-PCR assays using primers against ORF1b-nsp 14 and N genes	Sheridan, 2020; Udugama et al., 2020; WHO, 2020b
4	Novel coronavirus (2019-nCoV) Detection Kit	Amoy Diagnostics, China	Real-time RT-PCR method	Specific amplification of the ORF1ab and N conserved regions in viral RNA, and detection by fluorescent probes	AmoyDx, 2020; Sheridan, 2020; Udugama et al., 2020
5	RealStar® SARS-CoV-2 RT-PCR Kit 1.0	Altona Diagnostics, Germany	Real-time RT-PCR method	This method targets the detection of E gene in B-βCoV RNA and S gene in SARS-CoV-2 RNA using specific fluorescent probes, which enables the parallel detection of B-βCoV specific RNA and SARS-CoV-2 specific RNA	Altona, 2020; Sheridan, 2020
6	2019 novel coronavirus (2019-nCoV) by real-time RT-PCR	Charité – Universitätsmedizin, Germany	Real-time RT-PCR method	This method targets the amplification of RNA-dependent RNA polymerase (RdRP), E and N genes in viral RNA and their detection with specific fluorescent probes using the real time RT-PCR workflow	Corman et al., 2020; Sheridan, 2020; Udugama et al., 2020
7	Real-time fluorescent RT-PCR kit for detecting 2019-nCoV	BGI Group, China	Real-time RT-PCR method	Specific amplification of the ORF1ab regions in viral RNA, and detection by fluorescent probes	BGI, 2020b; Sheridan, 2020
8	Novel Coronavirus 2019-nCoV nucleic acid detection kit (cPAS, combinatorial probe-anchor synthesis sequencing method)	BGI Group, China	Metagenomics and RT-PCR method	This metagenomics sequencing kit is based on combinatorial Probe Anchor Synthesis. It is able to detect both known and novel microorganisms, enabling monitoring of evolution during transmission	BGI, 2020a; Pang et al., 2020; Sheridan, 2020
9	TaqMan 2019-nCoV Assay Kit	Thermo Fisher Scientific, United States	Real-time RT-PCR method	This method employs the amplification of specific region of ORF1ab, S and N genes in viral RNA followed by their detection using fluorescent probes. Human RNase P was used as positive control. It runs on Applied Biosystems 7500 RT-PCR system	Sheridan, 2020; ThermoFisher, 2020
10	2019-nCoV nucleic acid detection kit	National Institute of Infectious Disease, Japan	Real-time RT-PCR method	This method involves the amplification of specific region of N gene in viral RNA followed by their detection using fluorescent probes	Nao et al., 2020; Sheridan, 2020; Udugama et al., 2020
11	Novel Coronavirus 2019 detection by Real Time RT-PCR	National Institute of Health, Thailand	Real-time RT-PCR method	Specific amplification of a region of N gene in viral RNA followed by their detection using fluorescent probes	Sheridan, 2020; Udugama et al., 2020; WHO, 2020c
12	On-site rapid molecular diagnostic system based on Shenzhen Shineway Technology	Hong Kong University of Science and Technology, Hong Kong	Real-time RT-PCR method	Integrated microfluidic PCR test employing silicon-based micro-heater module for rapid heating and processing of test samples	Sheridan, 2020
13	COVID-19 genesig Real-Time PCR assay	Primerdesign Ltd., United Kingdom	Real-time RT-PCR method	Specific amplification of a region of ORF 1ab gene in viral RNA followed by their detection using fluorescent probes	Primerdesign, 2020; Sheridan, 2020
14	QIAstat-Dx Respiratory 2019-nCoV Panel	Qiagen, Germany	Real-time RT-PCR method	Integrated sample prep and RT-PCR detection of 21 respiratory pathogens including 2019-nCoV; the result is analyzed in desktop QIAstat-Dx Analyzer	Sheridan, 2020
15	Biomeme COVID-19 Go-Strips	Biomeme, United States	Real-time RT-PCR method	This run on Biomeme’s mobile handheld qPCR devices available on the firm’s website	Biomeme, 2020; Sheridan, 2020
16	VereCoV^TM^ Detection Kit	Veredus Laboratories Pte Ltd., Singapore and Singapore Institute for Health Innovation, Singapore	Real-time RT-PCR method	It integrates an ultra-fast miniaturized PCR reactor for the amplification of target gene and a customized microarray to qualitatively detect 2019-nCov virus	Sheridan, 2020; Veredus, 2020
17	Tib-Molbiol’s 2019-nCoV Real-time RT-PCR kit	TIB Molbiol, Germany also via Roche Diagnostics, Switzerland	Real-time RT-PCR method	Specific amplification of RdRP or E or N genes in viral RNA, followed by their detection using fluorescent probes	Roche, 2020; Sheridan, 2020
18	TRUPCR COVID-19 Real-Time RT-PCR Kit	3B BlackBio Biotech India Ltd., India	Real-time RT-PCR method	Specific amplification of region of RdRP or E or N genes in viral RNA, followed by their detection using fluorescent probes	TRUPCR, 2020
19	PathoDetect COVID-19 Detection Kit	Mylab Discovery Solutions Pvt. Ltd., India	Real-time RT-PCR method	Specific amplification of viral genes in viral RNA, followed by their detection using fluorescent probes	Mylab, 2020
**(B) Serum-based test**					
1	anti-SARSr-CoV IgG and IgM ELISA kits	Chinese Academy of Sciences, China	Enzyme-Linked Immunosorbant Assay (ELISA)	Detection of antibodies in human serum produced against SARS-CoV-2 Rp3 nucleocapsid protein by ELISA	Udugama et al., 2020; Zhang W. et al., 2020
2	qSARS-CoV-2 IgG/IgM Rapid Test	Cellex Inc., United States	ELISA	Qualitative detection of IgM and IgG antibodies against SARSCoV-2 in serum, plasma or venipuncture whole blood from individuals suspected of COVID-19	Cellex, 2020
3	Diagnostic Kit for IgM/IgG Antibody to Coronavirus (SARS-CoV-2)	Zhuhai Livzon Diagnostics Inc., China	ELISA	Detection of IgM and IgG antibodies against SARSCoV-2 in serum, plasma or venous blood from individuals suspected of COVID-19	Udugama et al., 2020; Xiang et al., 2020
4	Novel coronavirus IgG/IgM antibody GICA kits	Zhuhai Livzon Diagnostics Inc., China	Colloidal Gold Immunochromatographic Assay (GICA)	Detection of IgM and IgG antibodies against SARSCoV-2 in serum, plasma, or venous blood using gold-labeled pad from individuals suspected of COVID-19	Udugama et al., 2020; Xiang et al., 2020
5	Peptide-based luminescent immunoassay to detect IgG and IgM	Key Laboratory of Molecular Biology on Infectious Diseases, Chongqing Medical University, China	Peptide based luminescent immunoassay	Detection of 2019-nCov IgG and IgM antibodies using synthetic peptide antigens from the orf1a/b, spike (S), and nucleocapsid (N) proteins as the immunosorbent	Cai X.-F. et al., 2020; Udugama et al., 2020
6	COVID-19 IgM-IgG Dual Antibody Rapid Test	BioMedomics, Inc., United States	ELISA	Qualitative detection of IgM and IgG antibodies against SARS-CoV-2 in serum, plasma, or venous blood from individuals suspected of COVID-19	BioMedomics, 2020
**(C) Non-invasive test**					
1	Low-Frequency Raman Spectroscopy as a Diagnostic Tool for COVID-19	Bar-Ilan University, Israel	Low-Frequency Raman spectroscopy	The distinct nanostructure of SARS-CoV-2 will give a unique spectral signature under low frequency Raman spectroscopy, which will not be decay by laser excitation	Jacobi et al., 2020

Serum-based diagnostic tests are generally antibody-targeted tools against either the whole SARS-CoV-2, or against Rp3 nucleocapsid (N) proteins, synthetic peptide antigens from the orf1a/b, S, and N proteins. Additionally, the non-invasive diagnostic test through which the viral infection can be quickly detected within 15 min has significantly gained attraction in the present situation and has been credited as a novel piece of work (Table 1A). Though these methods are effective in viral detection, some of which are even considered as gold standard for viral detection, like RT-PCR based techniques, these methods still possess their own pros and cons (Table 1B). However, the non-invasive methods of detection might be further enhanced with high accuracy by augmentation with nanotechnology (Kerry et al., 2019). There has been some evidence suggesting the efficacy of viral detection through nano-based viral sensors, which needs further improvement(s). But the potential associated with the application of nano-based materials is beyond the comprehension of our imagination (Kerry et al., 2018; Islam et al., 2019; Saylan et al., 2019).

**TABLE 1B:** Merits and demerits of different diagnostic methods used for SARS-CoV-2 detection.

Sl. No.	Detection methods	Merits	Demerits	References
1	RT-PCR method	It is the most frequently used gold standard frontline test for COVID-19 that directly detects the presence of viral RNA	RT-PCR requires prior sequence data of the specific target gene of interest	Lauri and Mariani, 2009; Green et al., 2020; Udugama et al., 2020
		It is fairly quick, sensitive and reliable, capable of producing results in 4–5 h	Difficulties in RNA isolation from samples like sputum or environmental samples may give false-negative results especially when target particles are low in number	
		This technology is widely available and very common in research and medicine, and already in place to test for COVID-19	Since this method detects current infection so there is a possibility to miss patients who have cleared the virus. Unable to monitor the progress of the disease stages so failed to perform broad identification of past infection and immunity	
		This method detects current infections of disease, can determine who is currently infected and who is not in population	Sometime false positive results may occur due to non-specific amplifications, so need highly skilled expertise in designing the kit components and detection	
2	Serological method	Lateral flow immunoassays are simple devices and easy to read that can detect antibodies (IgM/IgG) in the blood for both current and past infection of COVID-19	Unlike RT-PCR and ELISA, Lateral flow tests are time consuming and more expensive for large number of samples. As this technology is new, its reliability is still under evaluation	Cai X.-F. et al., 2020; Green et al., 2020; Udugama et al., 2020; Xiang et al., 2020
		This kind of test helps in epidemiology and vaccine development, and provides an estimation of both short-term and long-term track of antibody response, antibody abundance and its diversity	Serological assays will not be able to detect patients immediately upon infection until the immune system against virus is detected in blood	
		It is economical in terms of time (15 min to 2 h) and cost than RT-PCR, and techniques like ELISA is well established within science and medicine	Despite of hard work of several companies, ELISA tests are at preliminary stage for SARS-CoV-2 COVID 19 testing	
		ELISA can be used as rapid testing for multiple samples at once, so can be scaled up for large number of testing	Unknown	
3	Raman Spectroscopy	It is a cheap, quick and non-invasive virus detection method that relies on unique spectral signature of nanostructure of SARS-CoV-2	This method faces the challenge of fluorescence, background signals and laser intensity below ANSI limit	Desai et al., 2020; Jacobi et al., 2020; Khan and Rehman, 2020
		This method can be used for an *in vivo* point-of–care detection tool	This method cannot be used for vaccine development, and failed to track the antibody response and its abundance & diversity	
4	Nanotechnology-based techniques	The technique is quick, sensitive and reliable with higher accuracy and precision rate, low sample requirement, simple procedure	Cost-deficit and limited clinical experimentation	Baptista, 2014; Urio et al., 2015; Campos et al., 2020; Weiss et al., 2020
		The nanoparticles can be amalgamated to any of the classical technique for improvement of their detection efficacy		
		The nano-based techniques can be customized based on luminosity, chromogenic effect, acoustic effect, photothermal effect		

Researchers have been racing against time to understand this new virus and the etiology of this disease to unveil feasible treatment regimens and discover novel therapeutic interventions (Liu et al., 2020). There are no novel committed anti-viral therapeutics against COVID-19, but rather previously used antiviral strategies involving small molecules and biologics targeting complex molecular interactions are applied to control/check the infection and, in the meantime, scientists are tirelessly working to discover more effective methods. Some of the currently used conventional antiviral drugs are listed in Table 2. These drugs are conventionally used for treatment against other deadly viral or protozoal infections, such as HIV or AIDS, influenza virus, Ebola virus, respiratory syncytial virus (RSV), hepatitis C and B virus, RNA viruses like flaviviruses, togaviruses, bunyaviruses, arenaviruses, paramyxoviruses, coronaviruses, filoviruses, orthomyxoviruses, and picornaviruses, and helminthic and protozoal parasites (Table 2). Each of these drugs has been found to exert a certain degree of antiviral impact in SARS-CoV-2 infection. It has also been found that combinations of these antiviral drugs are effective for the treatment of COVID-19 patients (Stebbing et al., 2020).

**TABLE 2:** List of different possible antiviral drugs available for SARS-CoV-2.

Generic name	Conventional application	Mechanism of action (broad sense)	Effective evidences against Coronavirus	Side effects	References
Baricitinib (MW: 371.4 g/mol)	Applied against rheumatoid arthritis	***JAK inhibitor:*** Baricitinib reversibly inhibits Janus kinase 1, Janus kinase 2, Janus kinase 3 and Tyrosine kinase 2 (which belongs to the same enzyme family) via a signal transduction pathway involving STAT proteins which ultimately modulates gene expression in immunological cells	Richardson et al. (2020) identified that baricitinib can helps to reduce the infection in lung cells	• Promotes upper respiratory tract infections and cholesterol levels in blood • Develops herpes zoster, herpes simplex, urinary tract infections, and gastroenteritis	Winthrop, 2017; Richardson et al., 2020
Lopinavir (MW: 628.8 g/mol)	Applied with a combination of ritonavir to treat and prevent HIV/AIDS	***Protease Inhibitor:*** Lopinavir used as an antiretroviral protease inhibitor which applied with combination of other antiretrovirals. It is a peptidomimetic molecule and contains a hydroxyethylene scaffold which imitates the linkage of peptides of protease enzymes that targeted by the HIV-1 protease, thus preventing the activity of the HIV-1 protease.	Chu et al. (2004) reported lopinavir and ribavirin at the concentrations of 4 μg/ml and 50 μg/ml, respectively, is effective against *in vitro* antiviral activity against SARS associated coronavirus	• Causes diarrhea, headache, nausea, vomiting, stomach upset, drowsiness, dizziness, taste abnormality and trouble in sleeping	Chu et al., 2004; Lemmer and Liebenberg, 2013
Ritonavir (MW: 720.9 g/mol)	Applied to treat HIV infection	***Protease Inhibitor:*** Ritonavir considered as a HIV protease inhibitor which interact with cytochrome P450-3A4 (CYP3A4) that abundantly found in intestines and liver	Dong et al. (2020) found positive results when combined administrated with lopinavir at a ratio of 200: 500 mg/capsule (lopinavir: ritonavir, respectively)	• Helps to develop asthenia, malaise, diarrhea, nausea, vomiting, abdominal pain, dizziness, insomnia, sweating, taste abnormality, abnormality in metabolic activities	Zeldin and Petruschke, 2004; Dong et al., 2020
Darunavir (MW: 547.7 g/mol)	Applied to treat and prevent HIV/AIDS	***Protease inhibitor:*** Darunavir is a non-peptidic inhibitor of protease that lodges itself in the active site of protease through a number of hydrogen bonds. As a result, it is developing to increase interactions with HIV-1 protease	Harrison (2020) has proven the antiviral activity against COVID-19	• Induces diarrhea, nausea, abdominal pain, headache and body rash	Harrison, 2020
Favipiravir (MW: 157.1 g/mol)	Applied to fight against Influenza virus	***RNA polymerase inhibitor*:** Favipiravir is a pyrazinecarboxamide derivative which helps to convert the ribofuranosyltriphosphate derivative by host enzymes and selectively inhibits the influenza viral RNA-dependent RNA polymerase	Cai Q. et al. (2020) reported the effectiveness of favipiravir in 35 patients (out of 80) demonstrated significantly shorter viral clearance time, however, the exact mechanism is unknown. A regiment of 3200 mg (1600 mg twice daily) loading dose on day-1 followed by 1200 mg maintenance dose (600 mg twice daily) on day-2 to day-14	• Causes mild to moderate diarrhea, asymptomatic increase of blood uric acid and transaminases • Decreases the number of neutrophils	Cai Q. et al., 2020; Du and Chen, 2020; Shiraki and Daikoku, 2020
Remdesivir (MW: 602.6 g/mol)	Applied against Ebola virus	***RNA polymerase inhibitor****:* Remdesivir is a prodrug that metabolizes into its active form, GS-441524, which is an adenosine nucleotide analog, interferes with the action of viral RNA-dependent RNA polymerase, and evades proofreading by viral exoribonuclease (ExoN), causing a decrease in viral RNA production	Wang M. et al. (2020) experimented on COVID 19 patients and found the EC90 value of remdesivir in Vero E6 cells was 1.76 μM, suggesting its effectiveness	• Increases enzyme levels in liver • Causes nausea and vomiting	Agostini et al., 2018; Boettler et al., 2020; Wang M. et al., 2020
Ribavirin (MW: 244.2 g/mol)	Applied to treat RSV infection, hepatitis C and some viral hemorrhagic fevers	***Nucleoside inhibitor:*** Ribavirin is a guanosine analog which stops viral RNA synthesis and viral mRNA capping. In this way, it interferes with the RNA metabolism required for viral replication	Khalili et al. (2020) predicted the effectiveness of ribavirin individually or with the combination of either IFN-α/lopinavir/ritonavir. They had also stated the use of this drug during SARS and MERS outbreak	• Causes nausea, tiredness, chills or shaking, headache, mood changes, feeling irritable, muscle and stomach pain, vomiting and loss of appetite	Loustaud-Ratti et al., 2016; Khalili et al., 2020
Galidesivir (MW: 265.27 g/mol)	Applied against RNA viruses like Flaviviruses, Togaviruses, Bunyaviruses, Arenaviruses, Paramyxoviruses, Coronaviruses, Filoviruses, Orthomyxoviruses and Picornaviruses	***Nucleoside RNA polymerase inhibitor:*** Galidesivir works by binding to viral RNA polymerase where the natural nucleotide would bind, leading to a structural change in the viral enzyme due to altered electrostatic interactions	Elfiky (2020) predicted about galidesivir through molecular docking and postulated that it may tightly bind to the RdRp of the SARS-CoV-2 strain. The drug is currently in advanced development stage under animal trial to combat multiple potential viral threats including coronaviruses	• No notable adverse effects	Vickers, 2017; Westover et al., 2018; Eyer et al., 2019; Elfiky, 2020
Arbidol (MW: 477.4 g/mol)	Applied against a number of enveloped and non-enveloped viruses	***Cell membrane endocytosis inhibitor:*** Arbidol helps to inhibit the entry of cell by blocking/fusing with host cell membrane	Dong et al. (2020) reported about the clinical trial of this drug with 200 mg, 3 times/day may help to reduce the infection, but exact interaction is unknown.	• Develops nausea, diarrhea, dizziness and elevated serum transaminase	Boriskin et al., 2008; Huang et al., 2017; Dong et al., 2020
Chloroquine (MW: 319.9 g/mol)	Applied to prevent or treat the malaria	***Inhibition of MATE1-mediated metformin transport by chloroquine:*** Chloroquine inhibiting the formation of hemozoin from the heme that are released by the digestion of hemoglobin. The free heme then lyses membranes and leads to arrest the parasite. It also directly involved various molecular pathways in lysosomal activity, autophagy and signal transduction	Wong et al. (2020) discussed the advice given by the National Health Commission of the People’s Republic of China mentioning a dose of 500 mg twice per day for no more than 10 days best suitable for adults. Vincent et al. (2005) experimented with the effectiveness of chloroquine by observing the nature of Vero E6 cells with respect to SARS-CoV infection	• Results loss of appetite, mild dizziness. mild diarrhea, clumsiness, mild headache, nausea and stomach cramps	Vincent et al., 2005; Wong et al., 2020
Nitazoxanide (MW: 307.28 g/mol)	Applied to treat various helminthic as well as protozoal parasites and viral infections	***Interference with host-regulated pathways:*** Nitazoxanide inhibits the replication of a broad range of other RNA and DNA viruses including respiratory syncytial virus	McCreary and Pogue. (2020) reviewed the application of nitazoxanide which supports a potent *in vitro* activity against SARS CoV-2, by observing certain characteristics of Vero E6 and LLC-MK2 cells. Rossignol (2016) concluded that this might be due to interference with host-regulated pathways which are involved in viral replication rather than virus-specific pathways	• Develops nausea, stomach pain, headache, discolored urine	Rossignol, 2014, 2016; McCreary and Pogue, 2020
IFN-α (MW: 19.5 kDa)	Applied against chronic hepatitis B (HBV) and hepatitis C virus (HCV) infections	***DNA synthesis inhibitor:*** IFN-α may inhibit the DNA synthesis in meningioma cells induced by platelet-derived growth factor and epidermal growth factor	Sallard et al. (2020) discussed the broad antiviral applications of IFN-α under *in vitro* and clinical trials’ data says it is more effective in the early stages of infection. It interferes with the replication machinery of viruses and slows down the metabolic processes	• Swells or other reactions may persist at the injection site • Causes headache, tiredness, nausea, vomiting, diarrhea, trouble in sleeping	Ragel and Jensen, 2003; Dong et al., 2020; Sallard et al., 2020

The main reasons behind the effectiveness of these drugs are their ability to transverse across the cellular membrane. Further, this impact is complemented by their other determined functions, such as Janus kinase (JKA) inhibition (Richardson et al., 2020), viral protease inhibition (Harrison, 2020; Shamsi et al., 2020), viral RNA polymerase inhibition (Elfiky, 2020), nucleoside inhibition (Khalili et al., 2020), cell membrane endocytosis inhibition (Wong et al., 2020), the inhibition of multidrug and toxin extrusion protein (MATE) 1-mediated metformin transport (McCreary and Pogue, 2020), and DNA synthesis inhibition (Sallard et al., 2020). The currently available drugs have a certain promising effect against SARS-CoV-2, but also present complications for the host, except for Galidesivir (Crasto, 2020; NIH, 2020). Therefore, the approach should be taken to either eliminate or neutralize the adverse impacts of these drugs or to amalgamate other multipotential technological adversaries, like nanotechnology (Islam et al., 2019; Saylan et al., 2019).

## Nanotechnological Advancement in Detection of Viral Infection

Transdisciplinary and progressive evaluation of nanotechnology in viral detection have transformed conventional diagnostic approaches by converging electronics and surface science to areas of biomedical sciences (Vaculovicova et al., 2017). Exploiting nanomaterials for engineering nano-based-biosensors could facilitate early detection, even with a low volume of the sample (Campos et al., 2020). Nanosensors are commonly equipped with a receptor, a transducer, and a detector consisting of a digital output (Saylan et al., 2018). The target molecule comes into contact with the receptor (Goode et al., 2015) and the biological detection component identifies the molecule via reaction. Then, the transducer converts changes to a signal quantified by the detector (Verma and Bhardwaj, 2015). These can be categorized into five classes, that include electrochemical (Eissa et al., 2015), optic (Saylan et al., 2017), piezoelectric (Battal et al., 2018), thermal (Yang et al., 2016), and magnetic-based nanosensors (Wang et al., 2016). These sensors have been widely investigated to meet the requirements for clinical diagnostics, consisting of high sensitivity and early detection of several diseases (Baptista, 2014). Viral infections, like HIV, are mostly diagnosed via enzyme-linked immunosorbent assay (ELISA), that detects if HIV antibodies such as IgM/IgG are present or not (Urio et al., 2015).

There are different types of nanoparticulated systems (inorganic, organic, and hybrid NPs/nanosystems) which are classified based on their composition and can be exploited to develop an advanced and versatile detection system. For example, Lee et al. (2013) reported an electrochemical method for detecting the direct electron transfer signal from the HIV-1 virus through exploiting nanotechnology. In this method, AuNPs were electrodeposited onto the indium tin oxide coated glass (ITO) electrode to provide a higher background charging current and good electron transfer kinetics. AuNPs-antibody fragments were immobilized and modified with the ITO electrode using the self-assembly method through gold–thiol interactions. The technique was successful in detecting virus like particles from 600 to 375 pg/ml. Inan et al. (2017) developed a microfluidic nanosensor for the detection of human papillomavirus (HPV)-16 E7 antibodies and observed identification down to 2.87 ng/ml. The nanosensor was validated in serum samples and it was shown that the nanosensor could be engaged as a pretesting tool for the diagnostics of broad-spectrum monitoring of HPV-associated cancer. A digital nanosensor to detect the Ebola virus was developed by Natesan et al. (2019). This nanosensor had a flow cell assay that caught specific antibodies with microarrayed recombinant antigens and a smartphone fluorescent reader for high-performance elucidation of results. They showed that the smartphone reader had a hardware which connected to the back of a smartphone and provided the user with an interface to handle the operation, communicate with cloud services and acquire test results, and also developed a safe cloud service for the tele-monitoring of results (Natesan et al., 2019). Carbon-NPs-modified screen-printed carbon electrode (SPCE)-based electrochemical biosensor strip for rapid and sensitive detection of the Japanese Encephalitis Virus (JEV) was developed by Lai et al. (2017). Hamdy et al. (2018) developed an ultrasensitive AuNPs biosensor functionalized with thiol-linked oligonucleotides, which could recognize conserved RNA standards of foot and mouth disease virus (FMDV). Recently, Chowdhury et al. (2019) also developed a pulse-triggered ultrasensitive electrochemical sensor for the detection of Hepatitis E virus (HEV). Here, the group engineered a specific anti-HEV antibody-conjugated to nitrogen- and sulfur-co-doped graphene quantum dots (Ab-N and S-GQDs) and AuNPs-embedded polyaniline nanowires as the electrode matrix of the sensor. A colorimetric assay that detects HIV infection based on gold nanoparticles along with functional split aptamers has been developed for the detection of HIV-1 Tat protein, which is a transactivator of HIV gene expression (Fatin et al., 2019). Currently, a selective ‘naked-eye’ colorimetric assay based on AuNPs for the detection of SARS-CoV-2 has been developed by Moitra et al. (2020). Here, AuNPs capped with antisense oligonucleotides (ASO) specific for the N-gene (nucleocapsid phosphoprotein) of SARS-CoV-2 were developed that agglomerated selectively in the presence of its target RNA sequence and demonstrated a change in its surface plasmon resonance (SPR). These investigations are practical examples that suggest the applicability of nanotechnology in the detection of SARS-CoV-2 in future research.

## Nanotechnological Advancement in Inhibition of Viral Infection

With an aim of constructing a next generation nano-delivery system, researchers have shifted their curiosity to organic nanomaterials from inorganic NPs. With an effort to comprehend the intricacy of the body and to elude multiple veneers of defense of the host, it is necessary to depend on the capacity of certain materials to interact with specific components, without altering the host’s homeostasis (Weiss et al., 2020). Though organic nanomaterials are preferred over inorganic NPs, their efficacy and applicability in research involving viral inhibition are scanty. In the last two decades, inorganic NPs have been widely investigated for viral inhibition. Some of these include the use of graphene oxide (GO), which can effectively inactivate pathogenic agents of avian influenza A virus H9N2 and hand-foot-and-mouth disease EV7I, at 56^o^C (Song et al., 2015). GO composites functionalized with cyclodextrin and loaded with a high amount of curcumin could significantly inhibit infection of respiratory syncytial virus (Yang et al., 2017). Further, Lee et al. (2016) observed that solid and mesoporous silicon NPs functionalized with mimetic glycosaminoglycan could inhibit the entry of herpes simplex virus type 1 and type 2 into host cells. Mesoporous silicon NPs modified with different surface groups could effectively bind with the viruses through hydrophobic interactions and reduced the ability of the virus to infect host cells (de Souza et al., 2016).

Presently, numerous investigations exploiting organic nanomaterials for engineering nano-based viral inhibiting agents are being carried out. Kondel et al. (2019) have shown the control-release of acyclovir encapsulated in solid lipid nanoparticles (SLN) in a BALB/c mice model infected with herpes simplex virus (HSV)-1. Lauster et al. (2020) engineered phage capsid NPs functionalized with a sialic acid ligand to match the binding sites of the homotrimeric hemagglutinin to block influenza A virus entry in BALB/c mice. Their research demonstrated that the phage capsid NPs were effective against the virus and had no immunogenic impact on the host. Moreover, Wang W. et al. (2020) recently developed a dual-targeting ferritin NPs vaccine that could elicit an immunological response against chronic HBV in C57BL/6 mice. Here, the ferritin NP vaccine was synthesized by conjugating purified SpyCatcher-preS1 (SC-preS1) fused to SpyTag–ferritin NP. The preS1 conjugated with ferritin NP vaccine could simultaneously bind and induce SIGNR1 + dendritic cells (which activate T follicular helper cells) and lymphatic sinus-associated SIGNR1 + macrophages (which can activate B cells). Recently, Hu et al. (2020) commented on the therapeutic prospective of nanomedicine and chloroquine against COVID-19.

## Future Prospects of SARS-CoV-2 Detection Through Nanotechnology-Based Approaches

**FIGURE 2:**
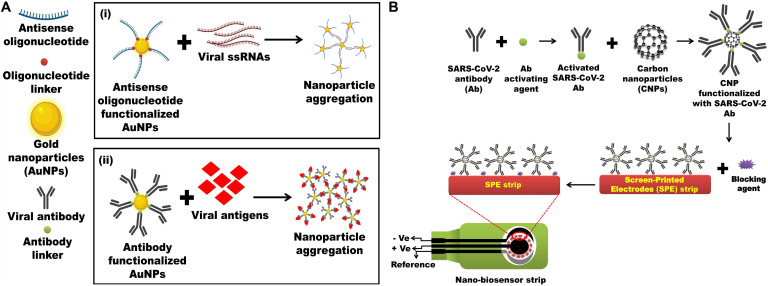
Nanotechnology-based detections of SARC-CoV-2 antigen or the whole virus. **(A) (i)** Multiple fragments of antisense-oligonucleotides against ORF1a/ORF1b/nsp3/nsp4/nsp5 functionalized on the surface of AuNPs. Antisense-oligonucleotides linked AuNPs binds with digested viral sample containing ORF1a/ORF1b/nsp3/nsp4/nsp5 genomic ssRNA, resulting in AuNPs aggregation which can be visualized spectrophotometrically as the color of solution changes from red to blue/purple. Inspired from Jacobi et al. (2020). **(ii)** Antibody against SARS-CoV-2 proteins such as S, E, and N protein and main proteinase (Mpro also called 3CLpro) functionalized on the surface of AuNPs will also aggregate after incubating digested viral sample containing SARS-CoV-2 proteins which can be visualized spectrophotometrically as the color of solution changes from red to blue/purple. Inspired from Jazayeri et al. (2018). **(B)** Screen-printed carbon electrode (SPCE) electrochemical biosensor strip can be designed against 2019-nCoV by exploiting the SARS-CoV-2 antibody immobilized onto the surfaces of carbon NPs (CNPs) through amide bonds formed between amino groups of CNPs and carboxylic groups of SARS-CoV-2 antibody. Inspired from Chowdhury et al., 2019.

Keeping these NPs-based biosensors as a reference, it is possible to develop nanobiosensors for the rapid and ultrasensitive detection of SARS-CoV-2. Hypothetically, an Au-NPs biosensor functionalized with thiol-linked oligonucleotides against ORF1a/ORF1b/nsp3 (viral protease) can be developed for the diagnosis of SARS-CoV-2 (Figure 2A). Optical properties of AuNPs are hugely considered and have the possibility for use in the recognition of chemical and biological materials (Jazayeri et al., 2018). A unique property of non-aggregate and aggregate modes of AuNPs is their ability to change color, which can be easily discriminated by UV spectrophotometer and can be effectively exploited for SARS-CoV-2 detection. Thus, in the current hypothesis, multiple fragments of antisense oligonucleotides against ORF1a/ORF1b/nsp3 can be functionalized on the surface of AuNPs. Then, these antisense oligonucleotides linked to AuNPs when added to a digested viral sample containing ORF1a/ORF1b/nsp3 genomic ssRNA, which will result in AuNPs aggregation. This can be qualitatively analyzed via spectroscopic methods as the color of solution changes from red to blue/purple (Figure 2A). Additionally, a carbon NPs electrochemical biosensor strip or a screen-printed carbon electrode (SPCE) electrochemical biosensor strip can be designed against 2019-nCoV by exploiting the SARS-CoV-2 antibody immobilized onto the surfaces of carbon NPs through amide bonds formed between amino groups of carbon NPs and carboxylic groups of SARS-CoV-2 antibodies. Similarly, for increasing the efficacy of the strip antibody against any of these viral proteins, like S, E, and N protein and main proteinase (Mpro also called 3CLpro), they can also be incorporated for the detection of the viral infection (Figure 2B). Again, surface proteins of SARS-CoV-2 could provide a unique spectral signature under low-frequency Raman spectroscopy, which will not be decayed by laser excitation. Therefore, a unique low-frequency Raman spectral signature of the whole virus or specific viral proteins can be generated and exploited as a novel identifier of the virus and viral protein components (Jacobi et al., 2020).

## Future Prospects of SARS-CoV-2 Inhibition Through Nanotechnology-Based Approaches

In addition to eliminating and/or neutralizing the adverse impacts of the antiviral drugs, it is complementary to devise a strategy for provoking or modulating the host cell intracellular cascades, like autophagy, inflammasome, apoptosis, and epigenetics, to act against the viral infection. The adverse impacts of these antiviral drugs are mostly due to their unambiguous target of action on both infected and uninfected cells, that occur at multiple locations of the host (Mohammadi Pour et al., 2019; Mishra et al., 2020). Nanotechnology-based advancements that have been made to combat these limitations are reviewed comprehensively by Kerry et al. (2019).

Taking into consideration these above-mentioned technological advancements made over the past decade, a nano-based antiviral drug and vaccine against SARS-CoV-2 can be proposed. To eliminate the adverse impact of antiviral drugs in COVID-19 patients, target-specific delivery of the antiviral drugs to goblet and ciliated cells are the basis, as these cells are the primary target of SARS-CoV-2 (Lukassen et al., 2020). Organic (polymeric/peptide) NPs encapsulating antiviral drugs can be functionalized with a goblet cell targeting agent, i.e., CSKSSDYQC peptide (Chen et al., 2018) and an antibody against dopamine-receptor type-5 (DR-5) [largely expressed in ciliated cells] for target-specific delivery of antiviral drugs (Pala et al., 2019). The efficiency of the hypothesized Targeted Organic Antiviral Nanosystem (TOAN) could be further enhanced by conjugating it with appropriate intracellular cascades (autophagy, inflammasome, and apoptosis) modulating agents.

**FIGURE 3:**
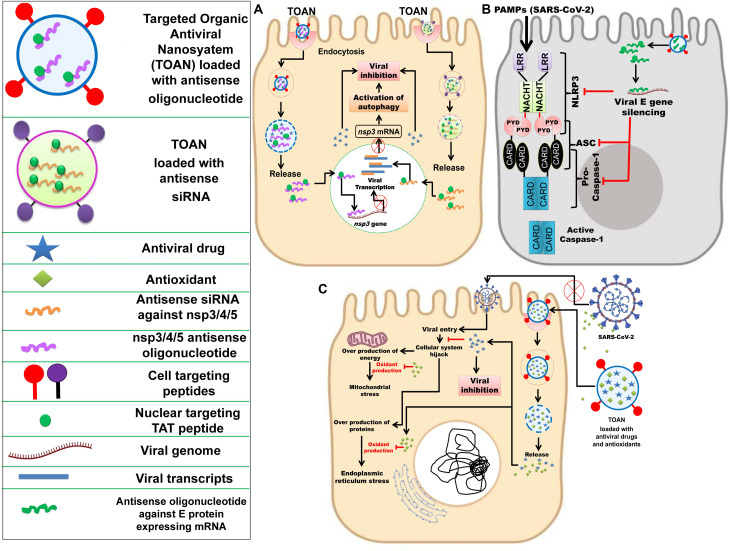
Nanotechnology-based inhibition mechanisms of SARC-CoV-2. **(A)** Delivery of nuclear targeting TAT peptide tagged antisense siRNA against nsp3/4/5 or TAT peptide tagged nsp3/4/5 antisense oligonucleotide along with an antiviral drug through targeted organic antiviral nanosystem (TOAN) to SARS-CoV-2 infected goblet cell, resulting in activation of autophagy and viral inhibition. Inspired from Pan et al. (2012); Chen et al. (2018), Panigrahi et al. (2018), and Pala et al. (2019). **(B)** Delivery of antisense oligonucleotide against SARS-CoV-2 E gene through TOAN to SARS-CoV-2 infected goblet cell, resulting in viral E gene and arrest of NLRP3. Inspired from Nieto-Torres et al. (2015); Siu et al. (2019), and Conti et al. (2020). **(C)** Delivery of antioxidants (flavonoids) and antiviral drugs through TOAN to SARS-CoV-2 infected goblet cell, resulting viral entry inhibition and destruction with complimentary antioxidant effect to neutralize mitochondrial and endoplasmic reticulum stress. Outside the infected cell, antioxidant compounds could effectively bind viral S protein and prevent its entry into the cell. Inspired from Galluzzi et al. (2017); El-Hamid et al. (2018), and Jena et al. (2020).

To effectively inhibit SARS-CoV-2, it is essential to identify the targeting intracellular viral components within the host, like various non-structural genes nsp3, nsp4, and nsp5. The nsp5 encodes 3CLpro which cleaves the replicase polyprotein pp1ab at the C-terminus at 11 sites, releasing nsp4-nsp16 that are essential in the viral life-cycle. The monomer of 3Clpro contains three domains and its active site that possesses a catalytic dyad of Cys145 and His 41 is located in between domain I and II (Wu C. et al., 2020). The chymotrypsin like protease plays an important role in processing pp1ab and viral replication (Mahdian et al., 2020; ul Qamar et al., 2020). As mentioned earlier, the nsp3-encoded PLpro enzyme cleaves the pp1ab at three distinct sites. In the case of coronavirus infection, it inhibits autophagosome to fuse with the lysosome and blocks the host’s immune response, promoting cytokine expression (Chen et al., 2014; Yin, 2020). For instance, in SARS-CoV-2-infected cells, activation of autophagy and viral inactivation can be simultaneously achieved by loading the TOAN with nuclear targeting TAT peptide tagged nsp3 antisense oligonucleotides/antisense siRNA (Pan et al., 2012; Panigrahi et al., 2018) (Figure 3A).

Further, the inhibition of the E protein may cause inactivation of inflammasomes as per Fung et al. (2020). But inflammasome initiation is mediated due to secretion of various inflammatory cytokines. However, the higher expression of cytokines can be reduced by inhibition of PLpro and this may subsequently inhibit inflammasome activation and blocking of the host’s innate immune response. Therefore, targeting PLpro along with 3CLpro could be a better therapeutic strategy. Siu et al. (2019) and Conti et al. (2020) highlighted the activation of NLPR3 inflammasome and secretion of IL-1β in SARS-CoV-infected cells, and previously Nieto-Torres et al. (2015) explained the virulent role of E protein toward the activation of NLRP3 inflammasome. Thus, to prevent NLRP3 inflammasome in the infected host cell, the TOAN can be conjugated with antisense oligonucleotides against SARS-CoV-2 E gene (Figure 3B).

The nsp4 is responsible for the formation of double membrane vesicle (DMV) and, likewise, other non-structural proteins are associated with several vital functions involved in virus maturation (Yin, 2020). Despite significant homology to SARS-CoV 3CLpro, the 3CLpro of SARS-CoV-2 is a conserved sequence due to some key differences in its substrate binding site. As 3CLpro is accountable for the processing of vital non-structural proteins (nsp4-nsp16), the conserved structure or its active site may serve as a target for antiviral therapies. Moreover, targeting 3CLpro may also prevent DMV formation due to the inactivation of nsp4 that may further inhibit ER stress because DMV is originated from ER of the host. Further, it is clear from previous studies that ER stress is one of the most well-regarded outputs of viral infection within the host cell. It is also understood that ER stress can have several deleterious effects on the maintenance of intracellular homeostasis, thereby compromising a cell’s ability to sustain and proliferate despite the infection, as well as perform its other regulatory functions as a part of a larger tissue system. Therefore, the exogenous reliving of intracellular ER stress by the utilization of natural antioxidants (flavonoids and terpenoids) would be complimentary with targeting nsp4 gene. Recently, computational studies have confirmed the ability of natural antioxidant flavonoids, like curcumin and catechin, to be proficient in binding and blocking the SARS-CoV-2 S protein (Galluzzi et al., 2017; El-Hamid et al., 2018; Jena et al., 2020). Hence, synthesizing TOAN in conjunction with these antioxidant flavonoids would be effective against SARS-CoV-2 (Figure 3C). A nano-based vaccine can also be designed against SARS-CoV-2 by exploiting the S protein, a conserved immunogenic epitope (Ou et al., 2020; Wang W. et al., 2020).

## Conclusion

The sudden emergence of SARS-CoV-2, along with its highly contagious nature, has affected countries and rattled societies throughout the globe. Researchers across the world have made advancements in characterization and are presently in search of effective detection and inhibition tactics. Therefore, this paper summarizes the available scientific knowledge of SARS-CoV-2’s structure and pathogenesis to better understand the virus. The paper also highlights the impaired and incomplete autophagy process induced by viral nsp3-encoded protease, enhanced ER stress, and apoptosis promoted through the generation of viral double-membrane vesicles, as well as posttranslational modifications of viral proteins. Further, the viral immunological response, envelope protein-mediated activation of NLRP3 inflammasome, and the genetic and epigenetic changes of host cells are also described. As a future prospective, the review has outlined two feasible colorimetric detection strategies that exploit AuNPs and nanosensors based on CNPs for the detection of SARS-CoV-2. Lastly, considering the requirement of an effective viral elimination method, three nano-based strategies have also been illustrated by using versatile organic nano-delivery system which need further investigation. Thus, the review provides a platform for present investigators to develop a competent method for SARS-CoV-2 detection and inhibition.

## Author Contributions

SP, RK, and SK wrote the initial draft. JR was credited for the first comprehensive edit, which was then reviewed and edited by GM and BP. All the authors read and approved the final manuscript.

## Conflict of Interest

The authors declare that the research was conducted in the absence of any commercial or financial relationships that could be construed as a potential conflict of interest.
